# Biomaterials-enabled cornea regeneration in patients at high risk for rejection of donor tissue transplantation

**DOI:** 10.1038/s41536-017-0038-8

**Published:** 2018-01-31

**Authors:** M. Mirazul Islam, Oleksiy Buznyk, Jagadesh C. Reddy, Nataliya Pasyechnikova, Emilio I. Alarcon, Sally Hayes, Philip Lewis, Per Fagerholm, Chaoliang He, Stanislav Iakymenko, Wenguang Liu, Keith M. Meek, Virender S. Sangwan, May Griffith

**Affiliations:** 1 Department of Clinical and Experimental Medicine, Linköping University, Linköping, Sweden; 2 Schepens Eye Research Institute and Massachusetts Eye and Ear Infirmary, Harvard Medical School, Boston, MA USA; 3 Filatov Institute of Eye Diseases and Tissue Therapy of the NAMS of Ukraine, Odessa, Ukraine; 4 Tej Kohli Cornea Institute, LV Prasad Eye Institute, Hyderabad, India; 5 Division of Cardiac Surgery, University of Ottawa Heart Institute, Ottawa, ON Canada; 6 School of Optometry and Vision Sciences College of Biomedical and Life Sciences, Cardiff University, Cardiff, UK; 7 Cardiff Institute for Tissue Engineering and Repair (CITER), Cardiff University, Cardiff, UK; 8 Key Laboratory of Polymer Eco-materials, Changchun Institute of Applied Chemistry, Chinese Academy of Sciences, Changchun, China; 9 School of Materials Science and Engineering, Tianjin University, Tianjin, China; 10 Department of Ophthalmology and Maisonneuve-Rosemont Hospital Research Centre, University of Montreal, Montreal, Canada

## Abstract

The severe worldwide shortage of donor organs, and severe pathologies placing patients at high risk for rejecting conventional cornea transplantation, have left many corneal blind patients untreated. Following successful pre-clinical evaluation in mini-pigs, we tested a biomaterials-enabled pro-regeneration strategy to restore corneal integrity in an open-label observational study of six patients. Cell-free corneal implants comprising recombinant human collagen and phosphorylcholine were grafted by anterior lamellar keratoplasty into corneas of unilaterally blind patients diagnosed at high-risk for rejecting donor allografts. They were followed-up for a mean of 24 months. Patients with acute disease (ulceration) were relieved of pain and discomfort within 1–2 weeks post-operation. Patients with scarred or ulcerated corneas from severe infection showed better vision improvement, followed by corneas with burns. Corneas with immune or degenerative conditions transplanted for symptom relief only showed no vision improvement overall. However, grafting promoted nerve regeneration as observed by improved touch sensitivity to near normal levels in all patients tested, even for those with little/no sensitivity before treatment. Overall, three out of six patients showed significant vision improvement. Others were sufficiently stabilized to allow follow-on surgery to restore vision. Grafting outcomes in mini-pig corneas were superior to those in human subjects, emphasizing that animal models are only predictive for patients with non-severely pathological corneas; however, for establishing parameters such as stable corneal tissue and nerve regeneration, our pig model is satisfactory. While further testing is merited, we have nevertheless shown that cell-free implants are potentially safe, efficacious options for treating high-risk patients.

## Biosynthetic corneas improve vision

A biomaterial implant supports the regeneration of severely damaged corneas in patients at high risk for rejecting conventional transplantation. An international team from Canada, China, India, Sweden, Ukraine and United Kingdom used mini-pigs to confirm the safety of implanting cell-free corneas made from recombinant human collagen and a synthetic lipid, before examining the effects of implantation on human vision in seven patients. The implants were well-tolerated and led to significant vision improvement in patients with damaged corneas due to infection. Furthermore, within two weeks of surgery the implants had relieved pain. Over two years, sensitivity to touch improved, suggesting an ability to promote nerve regeneration. This study supports the use of animal models to test biomaterials designed for medical applications and describes a safe and promising option for treating patients that not treatable by conventional corneal transplantation.

## Introduction

In tissue engineering and regenerative medicine, exciting new biomaterials and technologies such as 3D printing have produced very promising results in animal models, showing regeneration in a range of organs.^1^ However, translation of these remarkable accomplishments from animal models to patients in clinical practice has been protracted. One problem is the failure to obtain stable and functional integration of biomaterials into chronically damaged, diseased or aged tissues, unlike the case with mostly young, healthy animal models.^1^ The limited predictive power of pre-clinical animal studies, which typically involve the use of rodents and rabbits, has indeed been identified as the primary barrier to safe translation.^2^ More recently, pigs have been proposed as ideal pre-clinical models as the anatomy, physiology, and biochemistry of many of their organs,^3,4^ including their corneas,^5^ is similar to that of humans, allowing for greater predictability of performance of new implants in human subjects.

The human cornea is a relatively simple tissue comprising three main layers, an outer multilayered epithelium, a middle stroma consisting of a largely collagenous extracellular matrix and cells arranged in layers, and an inner single-layered endothelium. It is highly innervated but avascular, and is optically transparent to allow entry of light into the eye for vision. Irreversible loss of transparency can result in corneal blindness. Corneal blindness is estimated to affect 23 million individuals worldwide^6^ and is treated by corneal transplantation to restore clarity. However, there is a severe worldwide shortage of donor tissues, as with other transplantable organs. With only one donor cornea available for every 70 needed,^7^ it is evident that an alternative solution to just increasing the donation rate is crucial. Furthermore, corneas with inflammation and severe pathologies have a high risk (up to 49%) for rejecting conventional donor allografts.^6^ Over 90% of all cornea blind individuals and in particular, high-risk individuals, live in low to middle-income countries (LMICs).^6,8^ Due to a lack of resources in these countries, the treatment of these high-risk patients with stem cell technology is not possible, and the limited supply of donor tissues is prioritized for lower risk patients that have a higher chance of successful recovery.^9,10^ Artificial corneas known as keratoprostheses have been developed but only two have been successful in clinical use.^11^ However, because of the risk of severe side effects such as potentially blinding glaucoma, and the need for immune suppression and lifelong antibiotics, these are generally used only in end-stage diseased corneas.^12^ Full-thickness corneal grafting by penetrating keratoplasty (PK) remains the mainstay of corneal transplantation globally, particularly in LMICs^13^ even though partial thickness grafts that address only the affected layers are gaining in popularity. For damage affecting the epithelium and stroma, partial-thickness anterior lamellar keratoplasty (ALK) is performed. Given the magnitude of the problem with an estimated 1.52 million new cases of corneal blindness per year,^6^ cost-effective, cell-free biomaterials implants that promote endogenous regeneration while minimizing the regulatory and scientific challenges of specialized cleanrooms^14^ and immune rejection, are attractive clinical options.

Our team has previously shown that cell-free implants comprising carbodiimide-crosslinked recombinant human collagen type III (RHCIII) stimulated stable regeneration in conventional cornea grafted patients.^15,16^ However, for use in high-risk patients, we incorporated a second network of 2-methacryloyloxyethyl phosphorylcholine (MPC) as a structural element within the implant. MPC is a synthetic lipid reported to suppress inflammatory responses,^17^ and to minimize neovascularization in rabbit models of corneal alkali burn.^18^ In three pilot patients, the use of small tectonic patches of RHCIII-MPC to replace excised ulcerated tissue resulted in the successful restoration of corneal integrity without any adverse effects.^19^

Here, using the transplantation of RHCIII-MPC implants as a test bed, we assessed the efficacy of the pig model at predicting clinical outcomes. We examined the results of a pre-clinical mini-pig study alongside those of a clinical study involving seven high-risk patients with different pre-operative diagnoses. A pre-clinical study of biosynthetic corneas comprising RHCIII-MPC was performed in Göttingen mini-pigs to confirm previous safety results^20^ and to examine in detail the micro-architecture and optical properties of regenerated neo-corneas. For our clinical study, all recruited patients had been diagnosed with severe corneal pathologies resulting from ocular trauma or infective ocular ulceration and had consequently not been prioritized for grafting due to their high risk of donor rejection.^21^ The primary endpoint of the clinical study was safety, i.e., no serious adverse reactions such as excessive redness, pain or discomfort, swelling of adjacent corneal tissues or clouding of anterior chamber fluid. The secondary endpoints were the restoration of corneal integrity and regeneration of neo-corneas by mobilization of endogenous stem cells. The potential benefit to patients was the reduction of pain and discomfort to those with active ulcers, and the possibility of regaining eyesight in severely scarred eyes. The worst-case scenario was no change in vision or the need for re-grafting with a human donor cornea.

## Results

### RHCIII-MPC implants

RHCIII-MPC implants comprising 8% RHCIII, 4% MPC and 1.3% PEGDA were fabricated following Medical Devices Directive MDD 93/42/ECC and associated ISO standards in a certified and monitored cleanroom at Vecura AB, Karolinska University Hospital, Huddinge, Sweden. Only implants meeting quality control criteria such as comparable optical quality to the human cornea were used (Table 1A). Implants exhibited 92% light transmission,^22^ which is above the minimum of 87% for healthy human corneas.^23^ The refractive index of the implants was 1.334, similar to water (1.333) and marginally lower than that of the human cornea at 1.373.^24,25^ Denaturation temperature was 51 °C, lower than that of the human cornea at 65 °C^26^ but well above the highest body temperature ever recorded at 45 °C.^27^

**Table 1:** RHCIII-MPC implants and summary of pre-operative patient diagnoses: (A) Characteristics of corneal implants used in the study (*n* = 3); (B) Summary of pre-operative patient diagnoses

A				
Properties	Transmission in white light (%)	Refractive index	Denaturation temperature (°C)	Water content (%)
Implants	92.4 ± 0.1^22^	1.334 ± 0.0003^22^	51.0 ± 1.51	89.37 ± 2.1
QC acceptance criteria	≥85	1.1–1.5	≥50	≥85
Human cornea	87.1 ± 2.0^23^	1.373–1.380^24^	65.1^26^	78^25^

B							
Cause	Age	Gender	Stage of disease	Diagnosis	LESCD	Vascularised?	Start of disease until treatment (m)
Infection							
Patient 1	64	F	Acute	Herpes simplex keratitis	No	No	5
Patient 2	36	F	Scar	Fungal keratitis	No	No	8
Patient 3	56	M	Scar	Herpes simplex keratitis	No	No	72
Burns							
Patient 4	58	F	Acute	Alkali burn	Yes	Yes	480
Patient 5	73	M	Acute	Thermal burn (senile cataract in lens that impeded vision)	Yes	Yes	7
Other							
Patient 6	76	M	Acute	Rejected graft (no light perception due to glaucoma)	Yes	Yes	3
Patient 7	47	M	Acute	Neurotrophic keratitis	Yes	No	13

Other underlying, pre-existing conditions are shown in brackets

### Implants in mini-pigs

**Fig. 1:**
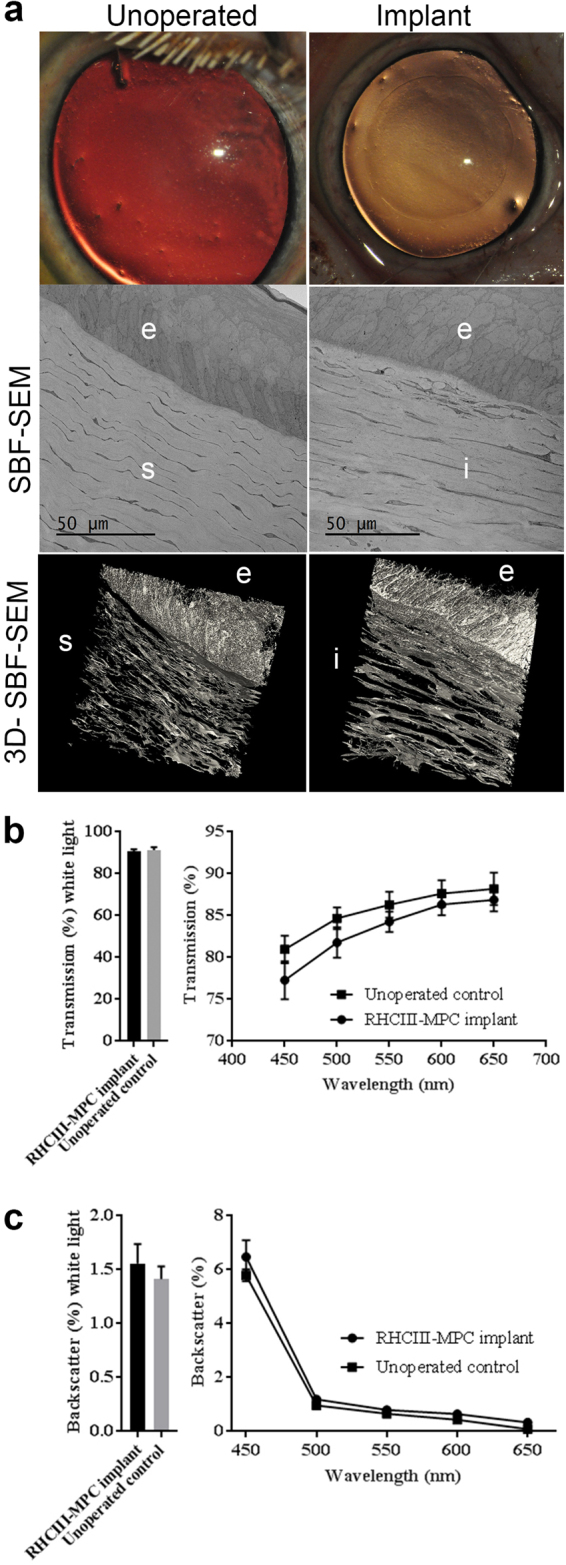
Regenerated corneas of Göttingen mini-pigs at 12-months post-grafting with RHCIII-MPC compared to healthy, unoperated control corneas. **a** control cornea versus RHCIII-MPC implanted cornea both showing comparable optical clarity. Serial block face-scanning electron microscopy (SBF-SEM) of single sections show that the epithelium is multilayered with comparable morphology including a layer of basal cells. Underlying the epithelium are stromal keratocytes arranged in lamellae. Scale bars, 50 µm. 3D reconstructions of the corneas show that both regenerated neo-cornea and healthy control comprise stromas with keratocytes arranged in highly ordered lamellae. **b** Light transmission profile of regenerated neo-corneas compared to healthy contralateral corneas. **c** Backscatter profile of regenerated neo-corneas compared to healthy contralateral corneas

RHCIII-MPC hydrogels (6.75 mm in diameter, 500 µm thick) were implanted the corneas of Göttingen mini-pigs by ALK, replacing the epithelium and anterior stroma but leaving the endothelium and posterior stroma intact. Implanted corneas were optically transparent like healthy, untreated control corneas at 12 months post-operation (Fig. 1a). In vivo confocal microscopical examination of the neo-corneas showed regenerated epithelium, stroma, and nerves (Supplementary Fig. 1), confirming the safety and efficacy reported in previous animal studies.^18,20^ Ultrastructural imaging using serial block face scanning electron microscopy (SBF-SEM) showed that both implanted corneas and unoperated controls had very similar epithelia and stromas. The epithelia in both had a well-defined layer of basal cells and layers of flattened interconnecting cells (keratocytes) were evident within the stroma (Fig. 1a).

As assessment of visual acuity or best-corrected visual acuity (BCVA) in mini-pigs was not possible, we examined the optical similarity of regenerated neo-corneas to that of healthy controls. Under white light illumination, the measured light transmission and backscatter values of the regenerated neo-corneas at 12 months post-operation did not differ significantly from that of the healthy, unoperated corneas (Figs. 1b, c; *P* > 0.05). Furthermore, when examined over a range of visible light wavelengths (450–650 nm), no significant differences in light transmission were detected between the operated and un-operated corneas (*P* > 0.05). However, the gradual drop in light transmission with decreasing wavelength, which was seen in both the operated and un-operated corneas, appeared to be slightly more pronounced in the regenerated neo-corneas (Fig. 1b). Values for percentage backscatter of light in the operated and unoperated corneas were almost identical over the entire visible light spectrum (Fig. 1c).

### Implants in patients

An open-label, first-in-human observational study was conducted following ISO 14971 - Medical devices—Application of risk management to medical devices, and ISO 14155:2011 - Clinical investigation of medical devices for human subjects—Good clinical practice. Clinical testing in Ukraine was performed in following the Declaration of Helsinki and relevant laws of Ukraine, following trial approval (ClinicalTrials.gov identifier NCT02277054) by the Bioethics Commission of the Filatov Institute of Eye Diseases and Tissue Therapy of the National Academy of Medical Sciences of Ukraine (FEI). In India, clinical testing was performed in accordance with the Declaration of Helsinki, relevant laws of India and after approval by the ethics committee (LEC 01-14-014) of the LV Prasad Eye Institute (LVPEI) and trial registration at Clinical Trial Registry-India (CTRI/2014/10/005114).

Seven unilaterally blind patients, aged 36 to 76 years old, diagnosed with conditions putting them at high risk of rejecting conventional corneal transplantation, and capable of providing informed written consent were recruited. Supplementary Table 1 shows the inclusion and exclusion criteria. Patients were divided into three groups based on the cause of corneal damage: infection, burns (chemical or thermal) and other (immune/degenerative disease) (Table 1B). The patients were also divided into two groups based on their stage of disease—acute phase (with ulcers and erosions) and post-scarring (severe scarring in need of scar revision). Before implantation, all acute patients suffered from chronic, recurrent episodes of pain accompanied by redness, photophobia, and tearing related to corneal de-epithelialization (Table 2B). Patients with severe scarring were asymptomatic.

**Table 2:** Patient outcomes after RHCIII-MPC implantation: (A) Clinical outcomes after RHCIII-MPC implantation at last follow-up; (B) Symptoms before surgery and at last follow-up after RHCIII-MPC implantation

A													
Patient no.	Graft diam. (mm)	Suture removal (weeks after surgery)	Full epithelial coverage (weeks after surgery)	BCVA pre-op LogMAR	BCVA at last follow-up, LogMAR	IOP at last follow-up in the operated eye (mmHg)	IOP at last follow-up fellow eye (mmHg)	Schirmer test at last follow-up operated (mm/5 min)	Schirmer test at last follow-up fellow (mm/5 min)	Corneal pachymetry Pre-op (µm)	Corneal pachymetry at last follow-up (µm)	Neovascularization of implant area at last follow-up	Follow-up (months)
1	6	8	4	LP	0·52	17	15	10	15+	250	470	No	24
2	8	3	12	1.6	0.1	14	12	16	20	484	270	No	35
3*	8	3	−	1.6	1·0	NA	NA	NA	NA	550	NA	NA	1.5
4	7	12	50	LP	1.7	13	14	15+	15+	1200	260	Yes	24
5	5	6	7	LP	1·3	19	18	6	10	320	320	Yes	14
6	4	12	48	NLP	NLP	25	15	14	14	410	560^**^	Yes	24
7	4	n/r	4	LP	1.3	16	16	9	8	220	1400	Yes	24

B						
No.	Photophobia / pain		Tearing		Redness	
	Before	After	Before	After	Before	After
1	+	−	+	−	+	−
2	−	−	−	−	−	−
3	−	NA	−	−	−	NA
4	+	−	+	−	+	−
5§	+	−	+	−	+	−
6	+	−	+	−	+	−
7	+	−	+	−	+	−

*Patient 3 dropped out of the trial due to an unrelated fungal infection that was treated by penetrating keratoplasty. His last follow-up examination was at 1.5 months post-operation

**18 months data

LP—light perception; *NLP* no light perception, *n/r* not removed

notes. ‘+’—symptom is present, ‘−’ —symptom is not present, ‘NA’—data not available

§Patient 5 did not come in for his 24 m follow-up but reported no symptoms when interviewed over the telephone by surgeon, OB at 24 m

Apart from one patient who was excluded from the study early on, all implants were well-tolerated over a follow-up period of 24 ± 6.6 months (range of 14–35 months) without immunosuppressive steroids beyond four weeks of prophylaxis, compared to up to 12 months of steroids for conventional PK allografts and even longer for high-risk grafts.

Clinical outcomes at the last follow-up varied depending on initial diagnosis (Table 2A). Patients with ulcers or scarring due to infection (Patients 1–3) showed the most improvement, followed by those with burns (Patients 4–5). The two patients with immune and degenerative disorders, i.e., previously rejected graft (Patient 6) and neurotrophic keratitis (Patient 7) performed most poorly.

Overall, epithelial cell migration over the implants took 4–50 weeks post-operation, being significantly slower in patients with diagnosed stem cell deficiency, but the healed ocular surface remained stable in all patients (Table 2A).

**Fig. 2:**
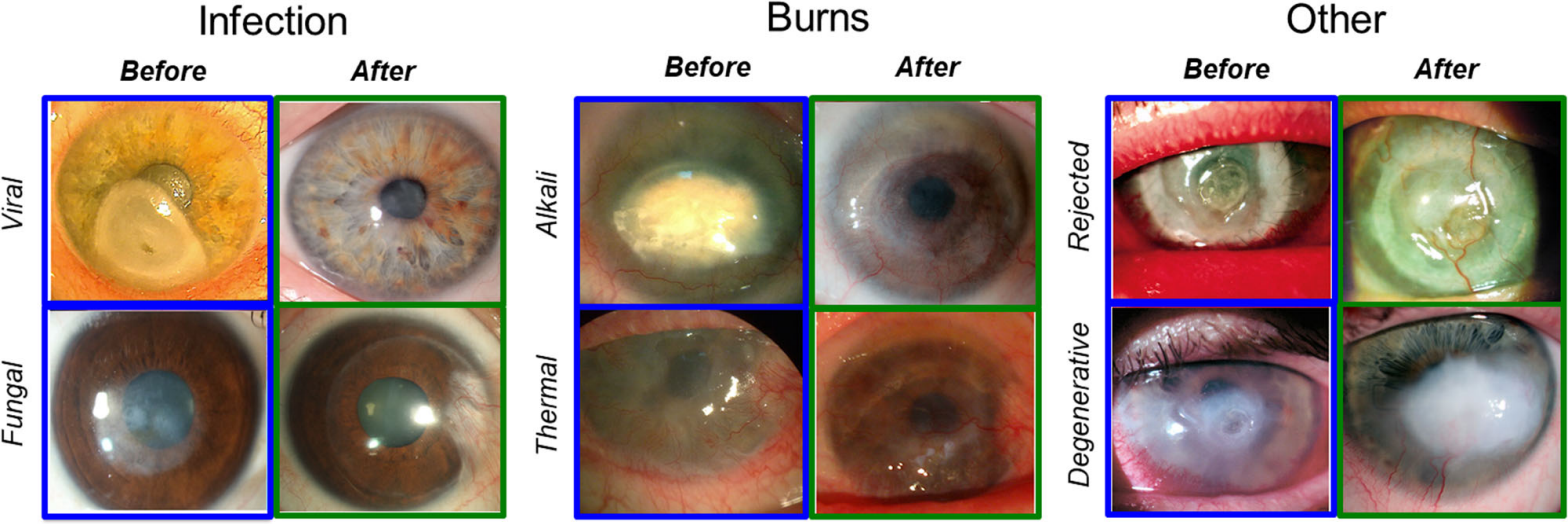
Patient corneas before and after grafting with RHCIII-MPC implants at last follow-up. Patients are divided into three groups based on their pre-operative diagnoses: infection (herpes simplex viral and fungal keratitis), burns (alkali and thermal) and other (failed graft and post-stroke neurotrophic keratitis). Post-operation, regenerated neocorneas from Patients 1 and 2 are mostly clear. In Patients 3 and 4, where stem cell deficiency is present, some superficial vessels concurrent with conjunctival invasion are seen. Patient 5 has a mostly clear cornea encircled by blood vessels but has invaded in one quadrant, while Patient 6’s cornea remains hazy. Patient 2 has an unrelated nasal pterygium

**Fig. 3:**
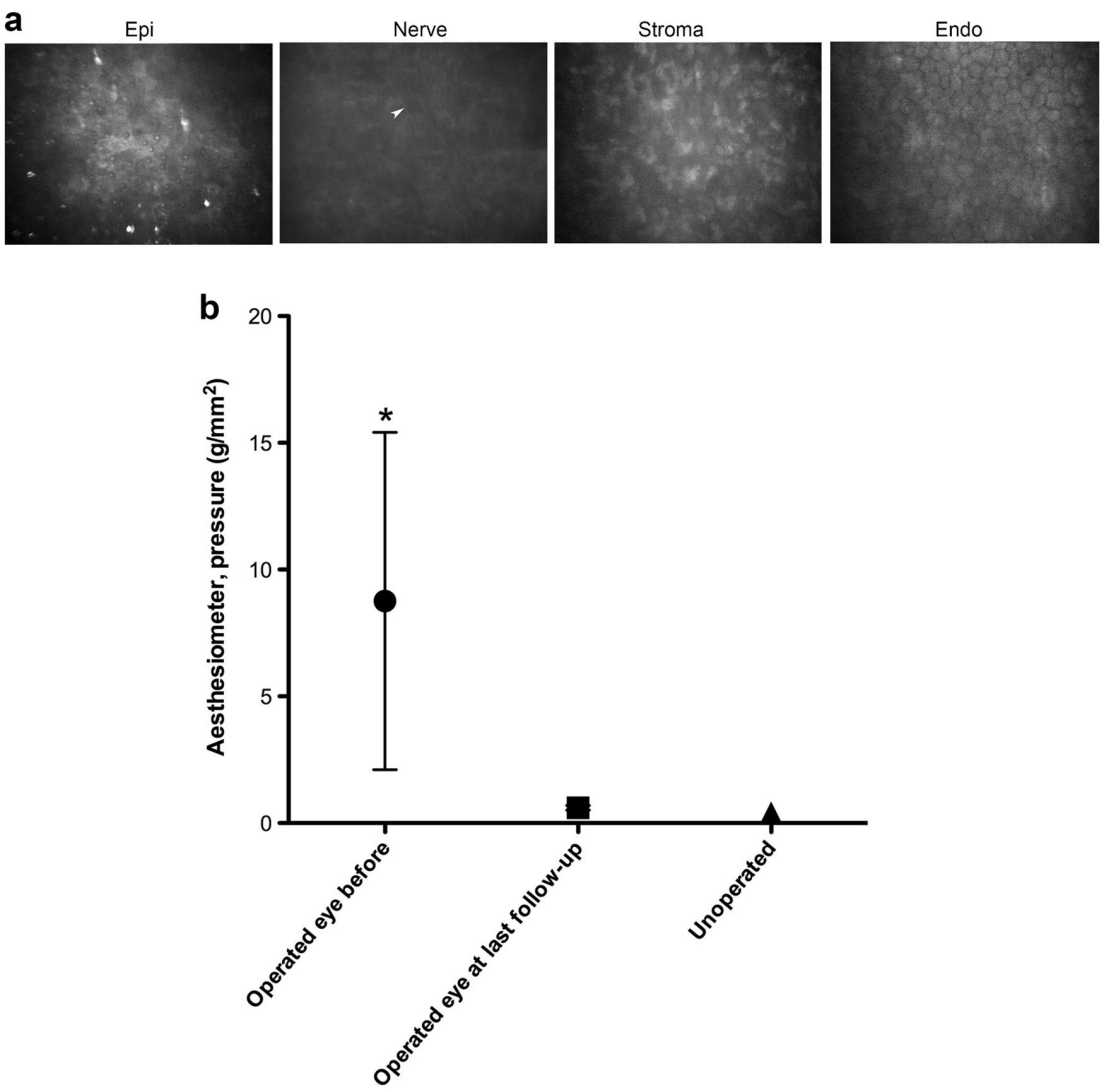
Regenerated patient corneas. **a** In vivo confocal images of the regenerated cornea from Patient 1 at 24 months post-operation, showing the regenerated epithelium, regenerating nerve (arrowhead) and stroma. The unoperated endothelium remains intact. **b** Changes in corneal touch sensitivity before and after RHCIII-MPC implantation as measured by Cochet-Bonnet aesthesiometry. The average pressure required to elicit a blink response from corneas before surgery, after implantation, and in comparison to the normal, healthy corneas. Touch sensitivity is inversely related to the pressure needed to elicit a blink response from the patients. Note: *p < 0.05 compared to unoperated contralateral eyes (Kruskal–Wallis test with Dunn’s correction for multiple comparisons)

In Patients 1–3, who had an ulcer or severe scarring due to infections, the implants were well-tolerated and stably incorporated. They remained relatively clear and edema-free (Fig. 2). Patient 1’s vision improved from near blindness (light perception) to 1.3 LogMAR at 2 weeks post-operation, to 0.7 LogMAR at three months and 0.52 LogMAR at eight months post-operation (moderate vision loss). Her vision remained stable over the 24 months follow-up period (Table 2A). In vivo confocal microscopical examination showed that she had fully regenerated epithelium and stroma, and her endothelium remains healthy (Fig. 3). A few corneal nerves were visible (Fig. 3). At 1-week post-operation, Patient 2’s vision had improved from 1.6 to 1.1 LogMAR. Complete epithelial coverage of the implant occurred over 12 weeks (Table 2A). The implant remained clear, free of edema and neovascularization. BCVA improved to 0.1 LogMAR (normal vision) by 6 months post-operation and remained stable over a follow-up period of 35 months. Ultrasound biomicroscopy and optical coherence tomography confirmed the preservation of the cornea curvature in Patients 1 and 2 that was restored by implantation (Supplementary Fig. 2). The vision of Patient 3 improved from 1.6 to 1.0 LogMAR at one-month post-operation (Table 2A). Unfortunately, he developed fungal keratitis at six weeks post-operation. Although this was deemed unrelated, as pathology findings showed the implant was untouched by fungus. However, the patient required therapeutic penetrating keratoplasty and was excluded from the study. All three patients had normal intraocular pressure (IOP).

In Patient 4, who had an alkali burn, healing was accompanied by implant thinning due to delayed epithelialization. Vision improved slightly from light perception to 2.0 LogMAR at 2 months and 1.7 LogMAR at last follow-up at 24 months, but the patient was still considered blind (Table 2A). Patient 5, who had a thermal burn pre-operatively, showed improved vision from near blindness (light perception) to 1.4 LogMAR at nine months post-operation and slightly decreased to 1.52 LogMAR (severe vision loss) at last follow-up at 14 months. It should be noted, however, that this patient also had a senile cataract that had progressed during the follow-up period, impeding vision despite an almost clear cornea. IOP was normal in both patients 4 and 5. Superficial vessels were seen in these corneas, which were previously vascularized and with limbal epithelial stem cell deficiencies (Fig. 2). These vessels were associated with invading adjacent conjunctiva. However, ultrasound biomicroscopy confirmed the preservation of the cornea curvature restored by implantation in both patients (Supplementary Fig. 2).

Patient 6 was blind due to glaucoma and had no light perception. He was grafted for symptom relief due to ulceration of a rejected corneal graft. Although the implant remained relatively clear, it was encircled by blood vessels, and these had invaded the implant margin at the 6–7 o’clock position. In Patient 7, the implant site was thickened considerably by epithelial hyperplasia causing the graft to become opaque, but the regenerated corneal tissue remained stable. The vision of Patient 7 was 1.4 LogMAR at 1-month post-operation, and despite cornea thickening remained stable throughout the follow-up and reached 1.3 LogMAR at last follow-up at 24 months post-operation. IOP was within the normal range for Patient 7 but not Patient 6 who had glaucoma before surgery (Table 2A).

Most notably, however, all five patients with acute disease and suffering from pain, irritation, and photophobia due to the ulceration, became asymptomatic within 1–2 weeks post-operation and remained as such at the last follow-up (Table 2B**)**.

Before surgery, all acute phase patients had reduced touch sensitivity from slight to the total absence of response (Fig. 3b). After surgery, sensitivity in all patients was restored close to levels observed in their healthy contralateral corneas, including the individual with degenerative neurotrophic keratitis (Patient 5). Kruskal–Wallis test showed a significant difference between average pre-operative sensitivity compared to contralateral eyes (*p* < 0.05).

## Discussion

RHCIII-MPC implants have been evaluated in a range of animal models including mice,^28^ rabbits^18^ and mini-pigs.^20^ The mouse implantation model is a rejection model and only provides information on implant performance relative to allografting.^28^ Rabbits have been used extensively in the pre-clinical testing of new corneal implants. For example, in a well-established alkali burn model that simulates severe pathology,^29^ we were able to show that the addition of the inflammation suppressing MPC to RHCIII biosynthetic corneas resulted in the implants remaining stably incorporated and clear.^18^ In contrast, burned corneas grafted with RHCIII only, like allografts, were vascularized.^18^ However, rabbit corneas differ from human corneas in that they are thinner, have no Bowman’s membrane and their endothelial cells proliferate readily.^30,31^ The pig cornea is structurally and mechanically closer to the human cornea,^5,32^ in that it possesses a Bowman’s membrane and an endothelium with minimal proliferative capacity.

In the present study, ultrastructural examination of the regenerated neo-cornea after RHCIII-MPC implantation in mini-pigs showed a micro-architecture that resembled that of healthy, unoperated corneas. The implants had stimulated the pig’s endogenous corneal progenitor cells to migrate into the implant, proliferate and recreate a neo-cornea. Optically, the regenerated neo-corneas also matched the healthy, unoperated corneas in allowing light transmission through the tissue with minimal back scatter.

As in the pre-clinical mini-pig study, the implants successfully stimulated endogenous cells to affect corneal repair in all of the six patients that completed the study. In most patients, the restored corneal surface led to improvements in BCVA that were maintained throughout the entire follow-up period of 14 to 35 months. The exception to this was Patient 6 who was blind due to glaucoma and therefore not expected to regain vision. All of the other patients showed vision improvement, but only two showed significant improvement from legally blind to vision impaired, and one gained normal vision. The vision of one of these patients was hampered by an unrelated senile cataract in his lens. Stem cell deficiency and conjunctival invasion were the main barriers to vision improvement. As the implants restored corneal stromal integrity, shown by the relief from pain, discomfort and photophobia, patients with stem cell deficiency can potentially undergo subsequent treatment to restore vision, e.g., if sufficient funding can be raised. Nevertheless, the healing and in-growth of cells to form neo-corneal tissue occurred and remained stable over the entire follow-up period, as seen in pre-clinical mini-pig model.

Epithelial coverage of RHCIII-MPC implants was significantly slower than reported for patients grafted with donor corneas by ALK or human amniotic membranes (HAM) to treat corneal thinning.^33^ The delayed epithelial closure is most likely due to retention techniques used^15^ as overlying sutures or excess glue retarded epithelial coverage creating an epithelial defect-like situation that most likely initiated an early inflammatory response. This was likely followed by induction of metalloproteinases and subsequent localized implant thinning. Corneal thickening due to epithelial hyperplasia, such as seen in one patient has been previously observed to a lesser extent in patients after refractive surgery^34^ and laboratory animals after corneal transplantation.^35^ Nevertheless, we have shown that RHCIII-MPC implants can be glued (albeit with caution with amounts used), opening up possibilities for future use as patches that may circumvent the need for transplantation.

A recent study comparing treatments for corneal ulceration showed that 100% of patients grafted with donor corneas by ALK became neovascularised.^33^ HAM grafts suppressed neovascularization but the membranes disintegrated within 6 months.^33^ Here, neovascularization was observed in the pre-operatively neovascularised corneas with limbal epithelial stem cell deficiencies. Neovascularisation concurrent with conjunctival invasion is common in corneas with limbal stem cell deficiency.^36^ These patients had severely damaged or scarred corneas so it is not surprising that their post-operative results and those of three earlier pilot patients^19^ were not as good as the outcomes seen here and elsewhere in healthy mini-pig corneas^20^ or even rabbit corneas after alkali injury.^18^ This discrepancy between animal data and clinical outcomes is typical when translating promising animal results into clinical application. Present results also show that recovery from a severe pathology has a different course to that seen in low risk patients.^11,15^ In low-risk patients, vision restoration was the indication for grafting but in high-risk patients, corneal surface restoration and symptom relief were the main indications for treatment of acute patients although vision improvement was the goal for scarred patients.

Very few therapeutic interventions promoting nerve regrowth into the cornea exist.^37^ In donor corneas grafted by ALK or PK, touch sensitivity remains low post-operatively.^37^ In our pre-clinical pig models, nerve regeneration was a main feature. We also noted regeneration of the different corneal nerve sub-types in guinea pigs grafted with collagen-MPC implants.^38^ Aesthesiometry performed on the acute phase patients showed that touch sensitivity, which is correlated with nerve function,^39^ was restored to near-normal levels in all five patients. Surprisingly, the functional restoration was also observed in Patient 7 who had no pre-operative touch sensitivity due to neurotrophic keratitis, a degenerative condition. HAM treatment has been reported to increase sensitivity in 9 out of 10 patients with similar profiles to our five patients.^40^ However, these patients had higher pre-operative sensitivity than our patients and their final sensitivity was just below normal. HAM contains a high concentration of growth factors and likely trophic factors that suppress inflammation,^40^ while the MPC used in our implants has reported anti-inflammation effects.^17^ Taken together, both observations strongly suggest that suppression of persistent inflammation in chronically ulcerated corneas facilitated nerve regeneration.

Following corneal wounding, elaboration of disorganized, unaligned mainly type III collagen occurs to form a scar.^41^ Here, bridging the wound gape with an organized matrix comprising aligned type III collagen,^22^ however, appears to provide a template for controlled in-growth of stromal cells that in turn allows for regeneration of an optically clear cornea. Furthermore, complications such as graft rejection (45% in high-risk patients) are likely elicited by the vascularized or inflamed host cornea reacting against the presence of allogeneic cells,^42^ were circumvented by use of cell-free implants. The inflammation inhibiting MPC networked within the implants most likely contributed to the capacity of RHCIII-MPC implants to remain quiescent in the immunogenic corneas, allowing stable restoration of the ocular surface.

It would also be pertinent to mention that even though stem cell replacement is an option in more affluent settings, there is still an issue with allogeneic transplantation that has not been solved. Systemic immune suppression is required for allografted corneal limbal epithelial cells, with reported severe side-effects that include anemia, hyperglycemia, elevated creatinine, and elevated levels of liver function markers.^43^ Furthermore, if the damage extends into the stroma, as seen in our patients, a second surgery requiring a human donor cornea is still needed.^43^ Here, the cell-free implants stimulated endogenous stem cells to affect the repair in both stroma and epithelium, together with nerve regeneration without immune suppression beyond prophylaxis.

With clinical application as the goal, synthetically-produced recombinant human collagen was used to circumvent immunogenic reactions that can occur with animal-derived collagen in susceptible patients due to their non-human protein composition,^44^ and pathogen transmission risks. Furthermore, our collagen-based biomaterials made for the cornea have been modified for use in other systems,^45–47^ as similar conditions such as skin ulcers in legs of diabetics, are enormous problems in LMICs.^48^

While confirmation in a larger number of patients is needed, we nevertheless demonstrate that implantation with cell-free RHCIII-MPC implants is a safe, reliable option for treating patients at high risk of donor allograft rejection; providing pain relief, and regenerating tissue and nerves. The clinical outcomes in humans although not as ideal as those in pre-clinical studies, nevertheless were predictable by use of wild-type mini-pigs as a model.

## Methods

### RHCIII-MPC corneal implants

European Medical Devices Directive MDD 93/42/ECC and its associated ISO standards were followed. For clinical evaluation, implants were made within an EU Class A laminar flow hood located in a Class B certified and monitored cleanroom at Vecura AB, Karolinska University Hospital, Huddinge, Sweden. Aseptic working conditions and sterile chemicals and reagents were used in the cleanrooms for implant production. Water for injection (WFI, HyClone, Utah, USA) was used to make up all solutions. For pre-clinical animal testing, implants were made under aseptic conditions in certified tissue culture hoods approximating Class A conditions.

Very briefly, implants were fabricated by mixing an 18% (wt/wt) aqueous solution RHCIII (FibroGen Inc., San Francisco, CA) with 2-methacryloyloxyethyl phosphorylcholine (MPC, Paramount Fine Chemicals Co. Ltd., Dalian, China) and poly(ethylene glycol) diacrylate (PEGDA, Mn 575, Sigma-Aldrich) in a morpholinoethane sulfonic acid monohydrate (MES, Sigma-Aldrich, MO) buffer. The ratio of RHCIII:MPC was 2:1 (wt/wt) and PEGDA:MPC was 1:3 (wt/wt). Polymerization initiators ammonium persulphate (APS; Sigma-Aldrich) and *N*,*N*,*N*,*N*-tetramethylethylenediamine (TEMED, Sigma-Aldrich) at ratios of APS:MPC = 0.03:1 (wt/wt), APS:TEMED (wt/wt) = 1:0.77, crosslinker, *N*-(3-dimethylaminopropyl)-N′-ethylcarbodiimide (EDC; Sigma-Aldrich) and its co-reactant, *N*-hydroxysuccinimide (NHS; Sigma-Aldrich) was then mixed in. The resulting solution was dispensed into cornea-shaped moulds and cured. After demoulding, they were washed thoroughly with phosphate buffered saline (PBS) and placed into vials of aseptic PBS containing 1% chloroform, which were sealed to maintain sterility.

During quality control, each implant was visually inspected for manufacturing flaws, discolouration or unwanted particulates under a dissection microscope. Those with imperfections were rejected. Batch controls were also performed on randomly selected implants, one from each batch (1 out of every 4 samples). Implants tested were found sterile, with endotoxin levels below the requirement of <0.5 EU/ml cut-off requirement for implantable medical devices^49,50^ by a Swedish Medical Products Agency approved laboratory (Apotek Produktion & Laboratorier AB, Stockholm, Sweden). Implants were tested to ensure their flexibility. Refractive index measurements were made using an Abbe 60 series Refractometer (Bellingham & Stanley Ltd., Kent, UK) calibrated against a silica test plate of known refractive index at room temperature. Light transmission through implants was measured by a UV-VIS spectrophotometer (U-2800 UV-VIS, Hitachi, Tokyo, Japan). Implant materials (5 × 15 mm) were placed within a quartz cuvette and positioned within the spectrophotometer in such a way that the beam was perpendicular to the hydrogel. Light absorption by the hydrogel was measured in the visual spectrum (400 to 700 nm). The equilibrium water content of hydrogels was determined to ensure uniformity. Hydrated hydrogels were removed from solution; the surface gently blotted dry and then immediately weighed on a microbalance to record the wet weight (*W*_0_) of the sample. The hydration of the hydrogels shown in Table S1 was calculated using a dry weight obtained by drying the samples at 60 degrees until constant mass was achieved (*W*). The equilibrated water content of the hydrogels (*W*_*t*_) was calculated according to the following equation: *W*_*t*_ = (*W*_0_–*W*) / *W*_0_ × 100%

The thermal stability of the implants was examined by measuring the denaturation temperature using a differential scanning calorimeter (DSC, Q20, TA Instruments, New Castle, UK). Heating scans were recorded in the range of 10 to 80 °C at a scan rate of 1 °C min^−1^. The samples ranging in mass from 3 to 5 mg were surface dried and hermetically sealed in pans. The denaturation temperature at the maximum of the endothermic peak was measured. Implants needed to pass both the visual inspection and batch sampling to be acceptable for clinical evaluation.

### Pre-clinical evaluation in mini-pigs

The study was carried out by Adlego Biomedical AB (Solna, Sweden), an approved GLP certified pre-clinical testing CRO. The methods performed were approved by the regional ethics committee (Stockholm norra djurförsöksetiska nämnd) and in compliance with the Swedish Animal Welfare Ordinance and the Animal Welfare Act and OECD Principle of Good Laboratory Practice, ENV/MC/CHEM (98) 17, 1997. Corneal implantation was performed in four female Göttingen SPF mini-pigs (Ellegaard, Denmark), 5–6 months old. Two weeks before surgery the animals were given a thorough clinical examination to establish a baseline for corneal health. During the surgery, the right cornea of each pig was trephined with a 6.5 mm diameter Barron Hessberg trephine to a depth of 500 μm. The corneal button was dissected lamellarly with a diamond knife and removed. A RHCIII-MPC implant, trephined to 6.75 mm in diameter was put into the wound bed. Human amniotic membrane (HAM; from the Cornea Bank, St. Erik’s Eye Hospital, Stockholm, Sweden) was placed over the implant to suppress undesired inflammation and the implants were held in place with overlying sutures. An antibacterial and anti-inflammatory ophthalmic solution (Tobrasone®, suspension with 3 mg/mL dexamethasone and 1 mg/mL tobramycine, Alcon, Sweden) was administered post-operatively. Each pig also received buprenorphine i.v. (0.05 mg/kg Vetergesic®, Orion Pharma, Finland). Subsequently, the operated eyes were treated 3 times daily with 1 drop Tobrasone®. Unoperated contralateral corneas served as controls.

Another four mini-pig implantations in Canada were approved by the University of Ottawa animal ethics committee (Protocol E-19) in compliance with the animal care guidelines of the Association for Research in Vision and Ophthalmology, and performed using similar methods. These corneas were used for measurement of optical properties.

At 12 months post-operation, after clinical data acquisition, the animals were euthanized. Both implanted and control corneas were harvested. Histopathological evaluation of GLP animals were performed by a Swedish MPA approved veterinary pathologist, BioVet AB (Sollentuna, Sweden). Optical properties such as % light transmission through the regenerated neo-corneas and the control unoperated eyes, and amount of back scattered light (%) were determined by measuring freshly excised corneas on a custom-built instrument equipped with a quartz halogen lamp for white light measurements as we previously reported.^51^

The mini pig corneal control and RHCIII-MPC implanted samples were fixed using 2.5% glutaraldehyde/2% paraformaldehyde in 100 mM cacodylate buffer pH 7.2 at room temperature for 12 h. The samples were then processed for the generation of high backscatter electron contrast for SBF-SEM as previously described.^52^ The samples were then transferred to a Zeiss Sigma VP FEG SEM equipped with a Gatan 3View2XP system, where data sets of 1000 images were acquired of the block surface every 100 nm through automated sectioning. Each image on the SBF-SEM was acquired at 4 K × 4 K pixels, at a pixel resolution 32 nm and a pixel dwell time of 8 µs, using an accelerating voltage of 3.4 keV in low vacuum variable pressure mode (28 Pa). Imaging data was acquired from a 134.93 µm × 134.94 µm region of interest. Selected serial image sequences were extracted from the image data and 3D reconstructions were generated using Amira 6.1 software (FEI Merignac, France).

### Patient surgeries and follow-up

At FEI, after providing written informed consent, patients were each grafted with a 350 µm thick RHCIII-MPC implant by ALK after manual excision of 300 μm of pathologic epithelium and stroma, except for Patient 2 who had a swollen, calcified cornea, and required excision of 900 µm of pathologic tissue. The excised pathological tissues, where available, were processed for histopathological examination. In two patients, only detritus was present so histopathology was not possible. The implants were retained by overlying sutures placed peripherally.^15^ After surgery, grafted patients received antibiotic eye drops (ofloxacin ophthalmic solution, 0.3%, Bausch & Lomb GmbH, Dr. Gerhard Mann chem.-pharm. Fabrik GmbH, Berlin, Germany) 4 times daily, short-term mydriatic (cyclopentolate 1%, Sentiss Pharma Pvt. Ltd., Gurgaon, India) and non-steroidal anti-inflammatory drug (Indometacin 0.1%, Bausch & Lomb GmbH) for 2 weeks, followed by topical antiseptic (chlorhexidine bigluconate 0.02%, Farmacia, Lugansk, Ukraine) and steroid (dexamethasone 0.1%, s.a. Alcon-Couvreur n.v., Puurs, Belgium) 3 times per day for the first week and tapered over 3 weeks to reduce post-operative inflammation. Patients wore bandage contact lenses until epithelial regeneration was complete. Sutures were removed between 3 and 12 weeks post-operatively in all patients except Patient 5, where the epithelium had grown over the sutures.

After providing written informed consent, LVPEI patients were each grafted with an 8 mm diameter, 350 µm thick implant by ALK after femtosecond laser (VisuMax, Carl Zeiss Meditec, Jena, Germany) assisted excision of 350 μm of pathologic epithelium and stroma. The implant was prepared using femtosecond laser and was retained using fibrin glue aided by overlying sutures placed peripherally.^15^ After surgery, patients were given moxifloxacin HCl ophthalmic solution 0.5% (Alcon, Fort Worth, USA) 4 times per day until re-epithelialization and topical 1 % Prednisolone acetate ophthalmic solution, (Allergan, Irvine, USA) 4 times per day for the first week and tapered over 3 weeks to reduce postoperative inflammation. Patients wore bandage contact lenses until re-epithelialization. Sutures were removed at 3 weeks post-operation.

All patients were assessed weekly until 1 month and then at 3 and 6 months, and then at 3–4 monthly intervals thereafter. Assessments of BCVA, IOP, tear production (Schirmer test), were made and slit-lamp microscopy was performed with and without fluorescein to confirm epithelial integrity. Patients also underwent ultrasound biomicroscopy (Aviso, Quantel Medical, Cournon-d’Auvergne, France) and in vivo confocal microscopy (ConfoScan4, Nidek, Japan) at FEI. At LVPEI, patients were examined by anterior segment optical coherence tomography (RTVue, Optovue Inc, Fremont, USA) to assess the cornea and anterior chamber. Nerve regeneration as evaluated by regaining of corneal touch sensitivity, was assessed using a Cochet-Bonet aesthesiometer (Luneau Oftalmologie, France). Very briefly, the aesthesiometer uses a 0.12 mm diameter nylon filament to obtain a blink response.^39^

### Statistical analyses

Quality control data in Table 1 are expressed as means ± SD. For optical properties measured for pig corneas, pairwise *t*-tests for white light and each wavelength was performed, with a Bonnferroni correction. Measurements of corneal nerve sensitivity were statistically evaluated using a Kruskal–Wallis test with Dunn’s correction for multiple comparisons. P values of <0.05 were considered significant.

### Data availability statement

Data analysed during the current study are included in this article and its supplementary information files. Clinical trial protocols are available through Clinicaltrials.gov (ID: NCT02277054) and the WHO International Clinical Trials Registry Platform (http://apps.who.int/trialsearch/; ID: CTRI/2014/10/005114). The individual patient data that support the findings are not publicly available due to patient confidentiality. Datasets generated are available from the corresponding authors (MG, KMM, NP, VSS) on reasonable request.

## Electronic supplementary material

Supplementary Materials
