# Supplementary material for: Biomaterials-enabled cornea regeneration in patients at high risk for rejection of donor tissue transplantation
Source: NPJ Regen Med. 2018 Jan 31;3:2. doi: 10.1038/s41536-017-0038-8 (PMC5792605; doi:10.1038/s41536-017-0038-8)
Supplement: Supplementary file 1 — Supplementary Materials [file 41536_2017_38_MOESM1_ESM.rtf]

SUPPLEMENTARY MATERIAL

Biomaterials-Enabled Cornea Regeneration in Patients at High Risk for Rejection of Donor Tissue Transplantation

M. Mirazul Islam, Oleksiy Buznyk, Jagadesh C. Reddy, Nataliya Pasyechnikova, Emilio I. Alarcon, Sally Hayes, Philip Lewis, Per Fagerholm, Chaoliang He, Stanislav Iakymenko, Wenguang Liu, Keith M. Meek, Virender S. Sangwan, May Griffith


Supplementary Table 1. Inclusion-exclusion criteria used in the recruitment of patients

Inclusion Criteria	Exclusion Criteria	
·	Adults, ages between 18 and 80 years, capable of giving written informed consent.
·	Legally blind in affected eye
·	Good vision in non-affected eye
·	Affected eye meets criteria of high risk for rejection and not prioritised for donor transplantation
·	Treatable by anterior lamellar keratoplasty (i.e. healthy endothelium)
·	Good general health	·	Systemic disease requiring medication
·	Perforated corneal ulcer.
·	Active  herpes  simplex   or  bacterial keratitis
·	Eye diseases  requiring other concurrent treatment regimens, e.g. glaucoma and severe dry eye disease.
·	Patients   with   poor   vision   in   their contralateral eye or active disease in their contralateral eye 
            that could lead to vision loss.
·	Eye    diseases    where    Descemet's membrane or corneal endothelium are involved.	


Supplementary Fig. 1.  In vivo confocal microscopical images of corneas of Göttingen mini- pigs at 12 months post-operation after grafting with cell-free RHCIII-MPC corneal implants. The regenerated neo-corneas show restored epithelia, sub-epithelial nerves (arrowheads), and stroma with keratocytes. The endothelium, which was unoperated, remains intact. In vivo confocal images from a healthy, unoperated cornea is shown as a control.


Supplementary  Fig. 2. Ultrasound  biomicroscopy  (Patients  1, 4-7) and anterior  segment- optical coherence tomography (Patient 2) images of corneas before and after RHCIII-MPC implantation.  Ulcerated  areas  in  patients  that  have  resulted  in  loss  of  stromal  tissue  are indicated by arrows. The arrowhead in Patient 2's pre-operative OCT image shows a large opaque scar (in white). Patient 4 has a very swollen cornea with an ulcerated surface indicated by the irregular superficial white margin. At last follow-up, the post-operative curvature of all the corneas show that restored surface integrity still remains.
